# A proof of concept study to assess the potential of PCR testing to detect natural *Mycobacterium bovis* infection in South American camelids

**DOI:** 10.1186/2046-0481-67-5

**Published:** 2014-02-07

**Authors:** Timothy R Crawshaw, Jeremy I Chanter, Adrian McGoldrick, Kirsty Line

**Affiliations:** 1 Animal Health and Veterinary Laboratories Agency Starcross, Staplake Mount, Starcross, Exeter, Devon EX6 8PE, England

**Keywords:** Alpaca, Llama, Mycobacterium bovis, Polymerase chain reaction, Clinical samples

## Abstract

**Background:**

Cases of *Mycobacterium bovis* infection South American camelids have been increasing in Great Britain. Current antemortem immunological tests have some limitations. Cases at post mortem examination frequently show extensive pathology. The feasibility of detecting *Mycobacterium bovis* DNA in clinical samples was investigated.

**Findings:**

A sensitive extraction methodology was developed and used on nasal swabs and faeces taken post-mortem to assess the potential for a PCR test to detect *Mycobacterium bovis* in clinical samples. The gross pathology of the studied South American camelids was scored and a significantly greater proportion of South American camelids with more severe pathology were positive in both the nasal swab and faecal PCR tests. A combination of the nasal swab and faecal PCR tests detected 63.9% of all the South American camelids with pathology that were tested.

**Conclusions:**

The results suggest that antemortem diagnosis of *Mycobacterium bovis* in South American camelids may be possible using a PCR test on clinical samples, however more work is required to determine sensitivity and specificity, and the practicalities of applying the test in the field.

## Background

*Mycobacterium bovis* infection in South American camelids (SAC) in Great Britain (GB) was first detected in a llama in 1999 [1] and the number of herds affected each year has shown an increasing trend [2]. The current ante mortem immunological tests available to detect TB in SACs do not have high sensitivity individually, though in if used in combination sensitivity can be increased [3]. In GB there is no routine testing of SACs for TB and as a consequence disease is often first detected only when a SAC dies and it is examined post-mortem. Cases at post-mortem examination frequently show extensive pathology and there is good concordance between pathology and culture for M. bovis [4]. In some herds disease has spread widely before detection, and losses in these herds can be very high. On post-mortem examination the characteristic gross pathology of TB in SACs can be extensive [5] suggesting that *M. bovis* may be present in nasal discharge, blood or faeces. While this could be a source for onwards spread of infection it could also facilitate ante mortem detection of disease.

A PCR test could offer improved specificity compared to immunological tests on blood. The calculated specificity of the antibody tests was 96.7 to 97.4%, with the sensitivity of the gamma interferon test specificity substantially lower [3]. A PCR test could be used on clinical samples from an unrestricted SAC herd, offering the possibility of detection of disease before there were SACs submitted for post mortem examination. A test with very high specificity would be more acceptable to the owners of SACs.

PCR testing does not appear to have been previously applied to clinical samples from SACs but PCR testing to detect members of the *Mycobacterium tuberculosis* complex have been applied to clinical samples in other species [6-10]. They have been used on human blood, sputum and faeces to detect *Mycobacterium tuberculosis*[6,8,10]. They have been used to detect *M. bovis* in bovine blood, nasal mucous and milk [7,9]. A PCR test has also been used to detect *M. bovis* in badger faeces and its use has been validated by an inter-laboratory trial [11].

The potential sensitivity of a test on clinical samples is dependent on the occurrence and quantity of the target organism in the clinical sample tested and the sensitivity of the test used. In the case of human blood, increasing levels of mycobacterial DNA have been found as the classification of disease severity increased [12]. The method of extraction at least for sputum appears to be critical to sensitivity [13].

The Regional Laboratories of the AHVLA examine carcases of SACs that have been euthanised as part of the Defra TB surveillance program. This provided an opportunity to collect clinical samples post mortem and examine them for *M. bovis*. A sensitive methodology for mycobacterial DNA extraction from faeces and nasal mucous using bead beating was developed and applied to the simulated clinical samples. The objective of this study was to determine if PCR testing using the sensitive extraction methodology on nasal swabs and faeces or directly on bloods could be used to detect *M. bovis* in SACs with a range of *M. bovis* pathology.

## Methods

Samples were collected from 63 SACs and all were from herds where infection with *M. bovis* had been confirmed by culture of tissues [10]. Samples were collected at four Regional Laboratories. Shortly after delivery of the carcase an attempt was made to collect a transthoracic cardiac blood sample. A nasal swab was taken before the carcase was hoisted to minimise contamination of nasal passages by leaking gut contents. The swab was passed 5 to 7 cms into each nostril. A faeces sample was taken from the rectum immediately on opening the carcass using clean gloves and instruments to minimise contamination. For each of the SACs sampled any pathology typical of *M. bovis* infection [5] was recorded in the post mortem report by the pathologists.

The samples were forwarded to the testing laboratory and nasal swabs and blood samples were partially processed prior to being frozen at -80°C, along with faeces, until DNA extraction and PCR were performed.

All the 63 SACs with samples were scored according to a pathology scoring system. The system scored them from no visible lesions of TB to extensive disease with extrathoracic spread. This was achieved by interpreting the post-mortem reports and scoring them according to the regime in Table 1. All of the parameters for all of the SACs were scored and the sum of the values assigned was the overall pathology score for that SAC. This system produced an ordinal scale with a minimum score of 0 and a potential maximum score of 12.

**Table 1:** Parameters used in the pathology scoring system

	**Value assigned**			
**Parameter**	**0**	**1**	**2**	**3**
Lung	No lesions	A few scattered foci	More extensive areas of caseation but not involving whole lung lobes	Severe and extensive caseation
Lungs affected	Neither lung affected	One lung affected	Both lungs affected	
Thoracic cavity	Pleurisy absent	Pleurisy present		
Liver	No lesions	Minimal lesions	More extensive lesions	
Mesenteric lymph nodes	No lesions	Lesions present		
Additional tissues affected	No additional tissues	1 additional tissue	2 additional tissues	3 or more additional tissues

Samples to take forward for PCR analysis were selected to be representative of the full range of pathology scores and included 8 SACs which had no grossly visible lesions of TB (Table 2). These samples came from 44 of the 63 animals examined. Where there were more animals available than required for testing the actual animals tested were selected randomly.

**Table 2:** Results of the nasal swab PCR, faeces PCR and a combination of the tests classified by pathology score

**Pathology score**	**Samples tested**	**Nasal swab PCR positive**	**Faeces PCR positive**	**Either test positive**
0	8	0	0	0
1	2	0	0	0
2	2	1	1	1
3	6	1	1	1
4	5	1	3	3
5	7	2	4	5
6	3	1	2	2
7	5	3	4	5
8	1	0	1	1
9	5	5	5	5
Total	44	14	21	23

Culture for mycobacteria [14] was carried out on selected tissues of fourteen of the 44 SACs tested in the study (Table 3).

**Table 3:** Results for the fourteen SACs where mycobacterial culture was undertaken

**Pathology score**	**Culture**	**Nasal swab PCR**	**Faecal PCR**	**Either pos**
0	*M. bovis*	Negative	Negative	Negative
0	Negative	Negative	Negative	Negative
0	Negative	Negative	Negative	Negative
1	Negative	Negative	Negative	Negative
2	*M. bovis*	Positive	Positive	Positive
3	Negative	Negative	Negative	Negative
3	*M. bovis*	Negative	Negative	Negative
4	*M. bovis*	Negative	Positive	Positive
5	Negative	Negative	Negative	Negative
5	*M. bovis*	Negative	Negative	Negative
5	*M. bovis*	Positive	Negative	Positive
6	*M. bovis*	Positive	Positive	Positive
7	*M. bovis*	Positive	Negative	Positive
9	*M. bovis*	Positive	Positive	Positive

### DNA extraction

#### Nasal swabs

Swabs were agitated into 0.2 ml phosphate buffered saline, centrifuged for 30 seconds at 16000 g in a microfuge to pellet cells, and the supernatant discarded. The pellet was resuspended in 0.1 ml. nuclease-free water. Samples were retained at -80°C. After thawing and the addition of 0.9 ml nuclease-free water, the samples were subjected to ‘bead beating’ to fully lyse cells present using a tissue homogeniser (Precellys 24, Bertin Technologies, France) and silica beads (Lysing matrix B tubes, MP Biomedicals, California). DNA was extracted from the lysate using an RNA/DNA extraction kit (MagMAX Pathogen RNA/DNA kit, Applied Biosystems, California) and magnetic particle processor (MagMAX Express 96, Applied Biosystems, California).

#### Faeces

2 g of faecal sample was first suspended in 35 ml sterile water overnight to fully hydrate the faeces and ensure release of bacterial organisms present. After 15 minutes of rocking and 30 minutes to allow settling of debris, a 20 ml aliquot of the top fraction was taken and centrifuged at 2,500 g, for 25 minutes. The supernatant was discarded and the pellet resuspended in 1 ml TE buffer (pH 8.0) and subjected to ‘bead beating’ as above. DNA was extracted as above.

#### Blood

A 0.5 ml aliquot of whole blood was retained for direct DNA extraction, and the remaining blood was processed to harvest the monocytes (buffy coat layer). The remaining blood was centrifuged at 2500 g for 10 min to fractionate the blood: Clear plasma (top), grey to green buffy coat layer (middle), and concentrated red blood cells (bottom). The plasma layer was removed and discarded. The buffy coat layer was carefully pipetted off the into a labeled Screw top vial. Both the whole blood and the buffy coat layer samples were retained at -80°C until DNA extractions were performed.

Both whole blood and Buffy coat samples were DNA extracted using a Qiagen QIAmp DNA Mini Kit, following the manufacturer’s instructions.

### PCR

#### IS1081 – TB complex

All samples were subjected to the IS1081 Real Time PCR to identify those positive for TB Complex DNA using a method based on that of Taylor, et al. [15] Primers 5′ CTGCTCTCGACGTTCATCGCCG 3′ & 5′ TGGCGGTAGCCGTTGCGC 3′ were used with probe [HEX] ATTGGACCGCTCATCGCTGCGTTCGC [BHQ1]. The target insertion sequence has multiple copies in the genome, offering good sensitivity.

#### RD4 – *M. bovis*

All samples positive for TB Complex IS1081 PCR were subjected to the RD4 Real Time PCR based on that of Taylor, et al. [15] to confirm the presence of *M. bovis* DNA. Primers 5′ TGT GAA TTC ATA CAA GCC GTA GTC G 3′ & 5′ ATG GCT ATT GAC CAG CTA AGA TAT CCG 3′ were used with probe 5′ [CY5] -CAA CAC TCT TGG AGT GGC CTA CAA CGG C [BHQ2] 3′. The RD4 deletion is a single target per genome and is expected to be of a lower sensitivity than the IS1081 PCR.

The PCRs were carried out using a PCR kit (QuantiTect Multiplex PCR NoRox kit, Qiagen, Germany) according to the manufacturer’s instructions on a real time PCR machine (Stratagene Mx3000P, Aglient Life Sciences, California). Both IS1081 and RD4 PCR were run for one cycle of 15mins at 95°C followed by 45 cycles of: 30 s at 94°C and 30 s at 60°C using HEX and CY5 probes respectively.

In addition to the 44 SAC samples, further DNA extraction control samples were used. A bovine faeces sample was used as a negative extraction control and a bovine faeces sample known to be positive for *Mycobacterium avium subsp. paratuberculosis* (MAP) was used as a positive extraction control. The DNA from the latter was subjected to Real Time MAP PCR to confirm successful DNA extraction.

The Cochran-Armitage test for a trend using statistical analysis software (StatXact software version 8, Cytel, Massachusetts) was applied to the proportion of positive results in the nasal swab and faecal PCR and a combination for the pathology scores.

## Results

The positive and negative extraction control samples were negative in the *M. bovis* PCR and MAP DNA was confirmed in the positive extraction control.

Samples from one llama and 43 alpacas were selected for DNA extraction and PCR. Forty sets of samples originated from one large outbreak with four sets of samples from three further outbreaks. Of the 44 SACs selected 8 had no visible lesions of TB and 36 had pathology scores between 1 and 9 on the ordinal scale (Table 2).

Some of the nasal swab samples were contaminated with blood as a result of leakage into nasal passages prior to samples being taken. All DNA extracts from samples were tested by IS1081 PCR, and 14 were found to be positive (mean CT value 31.1 range 24.3 to 38.8). The same 14 were all positive using the RD4 PCR (mean CT value 31.8 range 24.9 to 39.1), confirming the presence of *M. bovis*.

Of the 44 DNA extracts on faeces tested, 21 were positive for IS1081 (mean CT value 30.1 range 23.1 to 37.0) and again all 21 were confirmed as *M. bovis* by RD4 PCR (mean CT value 32.0 range 25.4 to 39.4).

Unfortunately suitable blood samples were only obtained from 3 SACs due to the difficulties of taking transthoracic blood samples post-mortem. The buffy coat DNA extraction samples yielded 1 sample positive for IS1081 of the three tested and which was also found to be positive by RD4 PCR. Whole blood DNA extracts did not yield any positive results.

The Cochran-Armitage test showed a highly significant increasing trend with pathology score in the proportion of positive results. The exact p-values calculated by for the nasal swab PCR or the faecal PCR alone and the combination of both were all <0.001.

Culture of tissue samples for mycobacteria was carried out on fourteen of the 44 SACs tested. Of the five that were negative on culture all were also negative in the PCR tests. Of the nine that were positive the faecal PCR did not detect five and the nasal swab PCR did not detect four. A combination of the PCR tests did not detect three (Table 3).

## Discussion and conclusions

The results of this study suggest that PCR testing of clinical samples from SACs may offer a route for the ante mortem detection of *M. bovis* infection. As the samples were taken post mortem these results may not replicate those that would be obtained with true clinical samples. More work including field validation is needed before routine use of this test could be considered.

While more SACs were positive in the faecal PCR problems associated with collection of the nasal swabs post mortem may have compromised the test on this sample type. A combination of the nasal swab PCR and faecal PCR appeared to have a marginally greater sensitivity than either of the tests alone. Testing of both sample types may increase sensitivity but at increased cost. Nasal swabs and faeces samples are relatively easy for owners to collect though there needs to be consideration of the possible exposure of owners to *M. bovis* when collecting nasal swabs. Pooling of samples from multiple SACs may potentially be a useful screening test. Too few blood samples could be collected for any evaluation.

There is a highly significant increasing trend in the proportion of positive results in the nasal swab and faecal PCR tests with increasing pathology score. This confirms, as expected, that a PCR test on clinical samples will have greatest sensitivity in SACs with more extensive pathology.

The number of SACs where culture results were available was small but the results were in agreement with the PCR results apart from three which were culture positive and PCR negative. This finding is not unexpected as post mortem and culture will be more sensitive than a PCR test on clinical samples.

Theoretically at least an uninfected SAC could ingest *M. bovis* contained in the secretions or excretions of an infected SAC that was in close contact. This might occur following contamination of feed in a trough for example. A very sensitive test might produce a positive result in these circumstances. In this study all the culture negative SACs were negative on PCR testing. In addition the gross pathology negative SACs which were also all negative on PCR had been housed in groups which had large numbers of positive animals. While this is a small study both of these findings suggest that contamination of uninfected alpacas by infected alpacas does not generate false positives in the PCR.

The gross pathology seen in SACs is frequently much more extensive than that seen in other species infected with *M. bovis*. This makes ante mortem detection of *M. bovis* in clinical samples less likely to be useful in species with less overt pathology. This is the most likely reason that PCR as a diagnostic tool in other species has been little investigated. Previous *M. bovis* PCR studies have been based on correlation with skin test results [7,9], not pathology of disease, or culture of tissue. The badger PCR work did not investigate the pathology of the animals, but the faeces alone [11].

Since the SACs tested were a biased sample selected to cover the range of pathology observed, it is not possible to accurately calculate a sensitivity. However, of the 36 pathology positive SACs, 23 were positive by PCR, providing a crude sensitivity of 64%. This compares to sensitivities of 57.7 to 69.2% for the antibody tests when used alone [3]. The sensitivity of the gamma interferon test depended on the parameters with the best sensitivities only possible with poor specificity [3]. The PCR test did not produce any false positive results, indicating a specificity of 100%. This compares to specificitiy of the antibody tests of 96.7% to 97.4% [3]. The PCR test offers the potential for a high specificity test with comparable sensitivity to the antibody tests.

More work is needed to demonstrate that it is practical to collect the clinical samples in the field. Testing of true ante mortem clinical samples both in infected herds and in herds with a low risk of TB are required to generate more data on sensitivity and specificity.

## Competing interests

The authors declare that they do not have any competing interests.

## Authors’ contributions

TC and KL participated in the conception and design of the study, and prepared the manuscript, AMG participated in the method development, and JC performed testing and acquired the data. All authors read and approved the manuscript.
